# Pharmacogenomic analysis of patient-derived tumor cells in gynecologic cancers

**DOI:** 10.1186/s13059-019-1848-3

**Published:** 2019-11-26

**Authors:** Jason K. Sa, Jae Ryoung Hwang, Young-Jae Cho, Ji-Yoon Ryu, Jung-Joo Choi, Soo Young Jeong, Jihye Kim, Myeong Seon Kim, E. Sun Paik, Yoo-Young Lee, Chel Hun Choi, Tae-Joong Kim, Byoung-Gie Kim, Duk-Soo Bae, Yeri Lee, Nam-Gu Her, Yong Jae Shin, Hee Jin Cho, Ja Yeon Kim, Yun Jee Seo, Harim Koo, Jeong-Woo Oh, Taebum Lee, Hyun-Soo Kim, Sang Yong Song, Joon Seol Bae, Woong-Yang Park, Hee Dong Han, Hyung Jun Ahn, Anil K. Sood, Raul Rabadan, Jin-Ku Lee, Do-Hyun Nam, Jeong-Won Lee

**Affiliations:** 1 Institute for Refractory Cancer Research, Samsung Medical Center, Seoul, Republic of Korea; 2 Research Institute for Future Medicine, Samsung Medical Center, Seoul, Republic of Korea; 3 Department of Biomedical Sciences, Korea University College of Medicine, Seoul, Republic of Korea; 4 Samsung Biomedical Research Institute, Samsung Medical Center, Sungkyunkwan University School of Medicine, Seoul, Republic of Korea; 5 Department of Obstetrics and Gynecology, Samsung Medical Center, Sungkyunkwan University School of Medicine, Seoul, Republic of Korea; 6 Department of Obstetrics and Gynecology, Dankook University Hospital, Cheonan, Republic of Korea; 7 Department of Neurosurgery, Samsung Medical Center, Sungkyunkwan University School of Medicine, Seoul, Republic of Korea; 8 Department of Health Sciences and Technology, Samsung Advanced Institute for Health Sciences & Technology, Sungkyunkwan University, Seoul, Republic of Korea; 9 Department of Pathology, Hwasun Hospital, Chonnam National University Medical School, Gwangju, Republic of Korea; 10 Department of Pathology and Translational Genomics, Samsung Medical Center, Sungkyunkwan University School of Medicine, Seoul, Republic of Korea; 11 Samsung Genome Institute, Samsung Medical Center, Seoul, Republic of Korea; 12 Department of Immunology, School of Medicine, Konkuk University, Chungju, Republic of Korea; 13 Biomedical Research Institute, Korea Institute of Science and Technology, Seoul, Republic of Korea; 14 Department of Gynecologic Oncology and Department of Cancer Biology, The University of Texas MD Anderson Cancer Center, Houston, TX USA; 15 Department of Systems Biology, Columbia University, New York, NY USA; 16 Department of Biomedical Informatics, Columbia University, New York, NY USA; 17 Department of Biochemistry and Molecular Biology, Ajou University School of Medicine, Suwon, Republic of Korea

**Keywords:** Gynecologic malignancy, Pharmacogenomic analysis, PARP inhibitor, *TP53* mutations, ID2

## Abstract

**Background:**

Gynecologic malignancy is one of the leading causes of mortality in female adults worldwide. Comprehensive genomic analysis has revealed a list of molecular aberrations that are essential to tumorigenesis, progression, and metastasis of gynecologic tumors. However, targeting such alterations has frequently led to treatment failures due to underlying genomic complexity and simultaneous activation of various tumor cell survival pathway molecules. A compilation of molecular characterization of tumors with pharmacological drug response is the next step toward clinical application of patient-tailored treatment regimens.

**Results:**

Toward this goal, we establish a library of 139 gynecologic tumors including epithelial ovarian cancers (EOCs), cervical, endometrial tumors, and uterine sarcomas that are genomically and/or pharmacologically annotated and explore dynamic pharmacogenomic associations against 37 molecularly targeted drugs. We discover lineage-specific drug sensitivities based on subcategorization of gynecologic tumors and identify TP53 mutation as a molecular determinant that elicits therapeutic response to poly (ADP-Ribose) polymerase (PARP) inhibitor. We further identify transcriptome expression of inhibitor of DNA biding 2 (ID2) as a potential predictive biomarker for treatment response to olaparib.

**Conclusions:**

Together, our results demonstrate the potential utility of rapid drug screening combined with genomic profiling for precision treatment of gynecologic cancers.

## Background

A fundamental principle of precision oncology is that molecular profiling of the tumor enables identification of appropriate therapeutic choice for individual patients [1–8]. However, predicting successful therapies on the sole basis of computational approach remains challenging [9–11]. Large-scale pharmacogenomic analyses using conventional cancer cell-line models have shown significant conceptual advances in discovering alternative therapeutic options for subsets of cancer patients [12–18]. However, molecular and pharmacological discrepancies between patient tumors and long-term cultured cancer cell-lines discourage clinical application of current gene-drug atlas. We have previously established a pharmacogenomic landscape of patient-derived tumor cell (PDC) models to reveal unprecedented insights into dynamic gene-drug associations and demonstrated its clinical feasibility [19]. To further interrogate the dynamics of pharmacogenomic interactions at single tumor-lineage resolution, we generated a collection of gynecologic tumors, including cervical, endometrial/uterine, and epithelial ovarian cancers (EOCs), and explored potential gene-drug associations against 37 molecularly targeted agents.

Currently, there are over 100,000 newly diagnosed cases and approximately 32,000 mortalities from gynecologic cancers in the US. Gynecologic tumors can be categorized into 5 distinct subgroups: ovarian, endometrial/uterine, cervical, vulvar, and vaginal tumors based on geographical locations. The current standard treatment consists of aggressive surgical intervention followed by platinum–taxane chemotherapy. Despite such intensive treatment modalities, approximately 25% of the patients invariably undergo tumor relapse within 6 months from the initial treatment and there is no alternative therapeutic avenue that is readily available. Although large-scale genomic characterizations of ovarian, uterine, and cervical cancers have been profiled by The Cancer Genome Atlas (TCGA) Research Network [20–23], clinical application potential of molecular targeted therapy remains obscure. Toward this goal, we have established a library of short-term cultured PDC models and performed comprehensive analyses of pharmacogenomic interactions to identify potential molecular determinants that could guide personalized treatment in gynecologic tumors.

## Results

### Establishment of patient-derived gynecologic tumor cell library

**Fig. 1:**
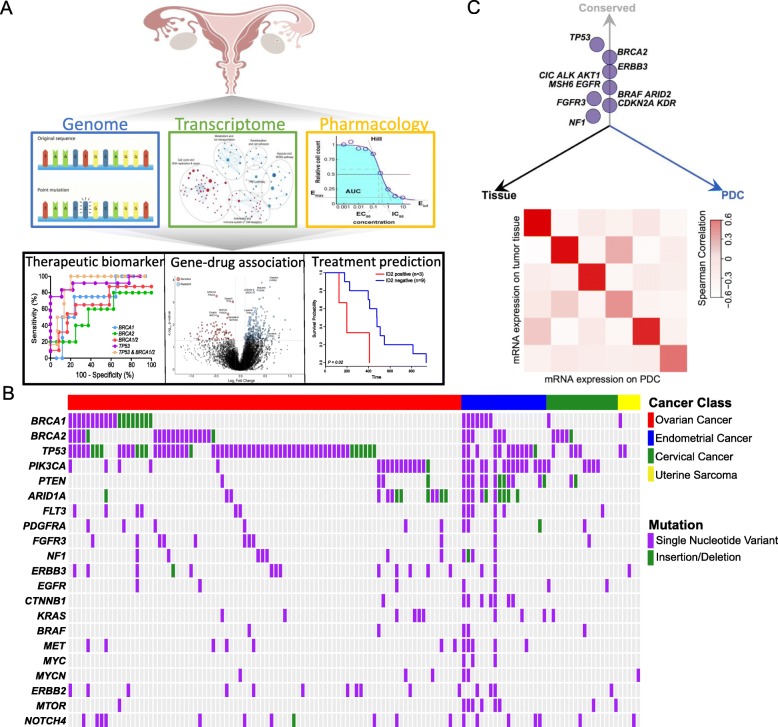
Pharmacogenomic analyses of gynecologic malignancies. **a** Schematic representation of pharmacogenomic analyses in gynecologic tumor-derived PDCs. Genomic and transcriptomics data were analyzed to identify single nucleotide variations and small indels and gene expression profiles. Short-term cultured PDCs were subjected to drug sensitivity screening against 37 molecular targeted compounds. **b** Mutational landscape of gynecologic tumors including ovarian cancer, endometrial cancer, cervical cancer, and uterine sarcoma. All mutations with an allele frequency of > 5% and depth of > 20 reads are shown. **c** Three-dimensional bubble plot demonstrating the frequency of non-synonymous cancer-driver mutations exclusively in tissue (black, left axis), PDC (blue, right axis), or shared between the two (gray, upper axis) (upper panel). The position of each dot or mutation is located on the quadrant based on its shared or private rate between primary tumor tissues and matched PDCs, and the distance reflects the number of cases that harbor respective mutation. Comparison of mRNA expression profiles between tumor tissue specimens and matched PDCs (bottom panel). Pearson’s correlation between tissue and PDCs is demonstrated as a heatmap

To establish a gynecologic PDC library, we have collected 139 tumor specimens from patients who were diagnosed with either cervical (CC) (*n* = 18), uterine/endometrial (*n* = 29), or epithelial ovarian (*n* = 92) cancers (Fig. 1a, Additional file 2: Table S1). Among them, 129 tumor tissues were subjected to targeted exome sequencing to identify genomic variations, including single nucleotide variants, short insertions and deletions, and copy number alterations [19, 24]. Somatic variants were determined though exome sequencing (median of 20 genomic variants per sample), and only mutations with variant allele frequency of > 5% were considered. Whole-transcriptome sequencing (WTS) was performed on 51 tumors to curate gene expression profiles. The mutational landscape of gynecologic tumors revealed enrichment of *TP53* somatic mutations in EOCs and endometrial cancers (EC) (Fig. 1b). *BRCA1* or *2* mutations were observed in 35%, 53%, and 38% of the sequenced tumors in ovarian, endometrial, and cervix cancers, respectively. Notably, genomic aberrations of Phosphoinositide 3-kinase (PI3K) pathway encoding genes including *PIK3CA* and *PTEN* were significantly more prevalent in endometrial tumors (*P* = 1.518 × 10^−06^ and *P =* 2.686 × 10^−08^, Fisher’s exact test; Additional file 1: Figure S1), suggesting potential therapeutic opportunities for PI3K targeted therapies [22, 25, 26]. Furthermore, somatic mutations of *CTNNB1* were predominantly observed in ECs compared with other gynecologic cancer types (*P =* 3.153 × 10^−03^, Fisher’s exact test) [22], indicating that targeting of Wnt/ß-catenin pathway could potentially provide clinical benefits for EC patients [27, 28]. Compared with TCGA datasets, our cohort constituted comparable levels of major cancer-driver genes in respective cancer types, including somatic mutations of *TP53* in EOCs, *PIK3CA* and *PTEN* in cervical cancers, and *PTEN*, *PIK3CA*, *ARID1A*, and *CTNNB1* in uterine corpus endometrial carcinomas (Additional file 1: Figure S2).

Tumor cell isolates were cultured under serum-free conditions for generally 2 to 4 weeks with epidermal growth factor (EGF) and basic fibroblast growth factor (FGF) supplements for enrichment of tumor initiating cell (TIC) populations (Additional file 2: Table S1) [29, 30]. Afterwards, PDCs were subjected to systematic drug sensitivity screening against 37 anti-cancer agents, targeting major oncogenic pathways including receptor tyrosine kinase (RTK), histone deacetylase (HDAC), and poly (ADP-ribose) polymerase (Additional file 3: Table S2 and Additional file 1: Figure S3) [19]. Drug sensitivities were determined using the area under curve (AUC) of the dose response curve (Fig. 1a) [19, 31–33]. A number of PDCs were further subjected to targeted exome sequencing and/or WTS to investigate whether the major gynecologic cancer-driver genes were retained from the parental tumors to PDCs. Consistent with previous observation, major drug-target genetic aberrations, including *TP53*, *ERBB3*, *EGFR*, and *BRAF*, were highly conserved in PDCs (Fig. 1c, Additional file 1: Figure S4). Moreover, transcriptome analysis of the parental tumors with matched PDCs demonstrated a strong positive correlation (Fig. 1c). To assess tumorigenicity of PDCs in vivo, we established patient-derived xenograft (PDX) models and evaluated their histological features [34]. Notably, PDX models recapitulated the original morphologic and pathologic characteristics of their parental tumors in situ (Additional file 1: Figure S5). Collectively, these results suggest that our gynecologic PDCs manifest molecular characteristics of the parental tumors and can be employed as surrogates for comprehensive pharmacogenomic analyses.

### Therapeutic landscape of gynecologic cancers reveals tumor type-specific drug sensitivity

Next, we established a pharmacological landscape of 66 PDCs that were derived from cervical (*n* = 6), endometrial (*n* = 10), uterine (*n* = 8, including leiomyoma), and EOCs (*n* = 42) using 37 molecularly targeted drugs. A total of 2442 drug-PDC combinations were analyzed and plotted (Additional file 4: Table S3 and Additional file 1: Figure S6). Distribution of drug sensitivities varied widely, portraying the heterogeneous nature of gynecologic PDCs. A subset of drugs, including afatinib, dacomitinib, neratinib (EGFR), AZD2014 (mTOR), panobinostat (HDAC), and trametinib (MEK), showed exceptionally high anti-tumor activities across all gynecologic tumors. In contrast, several agents, such as cabozantinib (VEGFR, MET, RET), ABT-888 (PARP), dabrafenib (BRAF), imatinib (Bcr/Abl), and sunitinib (PDGFR), demonstrated relatively minimal anti-cancer activities [19].

**Fig. 2:**
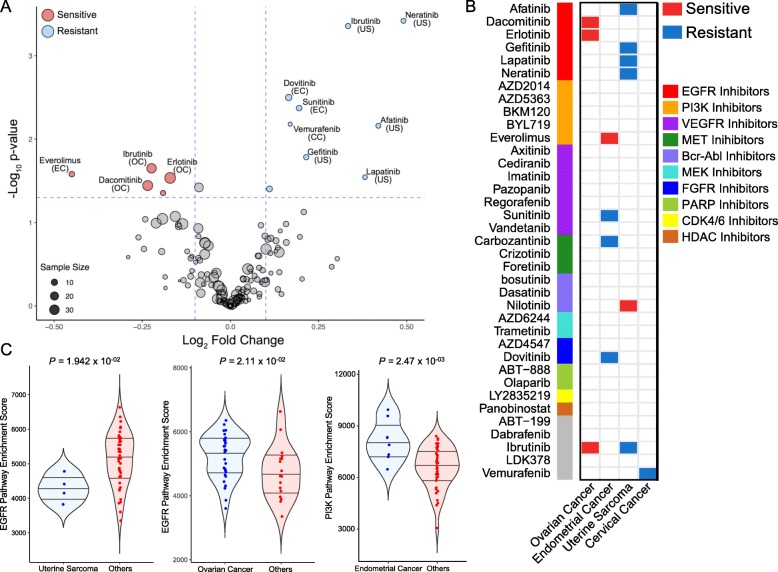
Lineage-specific drug sensitivity among gynecologic tumors. **a** Volcano plot representation of gynecologic tumor type-specific drug response, with fold-change drug comparison (*x*-axis) and its significance (*y*-axis). Each circle represents a single tumor type-drug interaction, and the size is proportional to the cohort size of the respective tumor. **b** Heatmap representation of plot A. Only significant associations have been marked based on sensitivity (red) or resistance (blue). Drugs have been clustered based on their known target classes. **c** Violin plots demonstrating the pathway enrichment scores of each corresponding pathway. The activity scores were measured using single sample Gene Set Enrichment Analysis (ssGSEA). Horizontal lines within the violin plots represent 0.25, 0.50, and 0.75 quantiles. *P* values in **a**–**c**: two-sided Wilcoxon’s rank-sum test

The molecular variations across diverse pathological tumor types could significantly contribute to distinct cancer type-specific drug response [12, 19]. Through lineage-specific drug sensitivity analysis (Additional file 5: Table S4), we discovered that EGFR inhibitors including erlotinib, dacomitinib, and ibrutinib, a BTK inhibitor that was previously shown to have profound anti-EGFR activities [19, 35], were highly sensitive in EOCs (*P* = 2.91 × 10^−02^, *P =* 3.59 × 10^−02^, and *P =* 2.24 × 10^−02^, respectively; Wilcoxon’s rank-sum test; Fig. 2a, b), whereas uterine sarcomas were considerably resistant to a number of EGFR-targeted compounds, including neratinib, afatinib, gefitinib, and ibrutinib (*P =* 3.81 × 10^−04^, *P =* 6.91 × 10^−03^, *P =* 1.65 × 10^−02^, and *P* = 4.39 × 10^−04^, respectively; Wilcoxon’s rank-sum test; Fig. 2a, b). Consistently, pathway enrichment analysis showed activation and downregulation of EGFR-associated pathway in EOCs and uterine sarcomas, respectively (Fig. 2c). Furthermore, we also discovered enrichment of PI3K pathway in ECs, which further advocated our previous observation on recurrent genomic aberrations of PI3K-AKT-mTOR (PAM) pathway in ECs (Fig. 1b) [29]. Consistently, everolimus, a mTOR inhibitor, exhibited significantly higher anti-tumor activities in ECs compared with other cancer types (Fig. 2a, b) [36].

### Pharmacogenomic interactions in EOCs

**Fig. 3:**
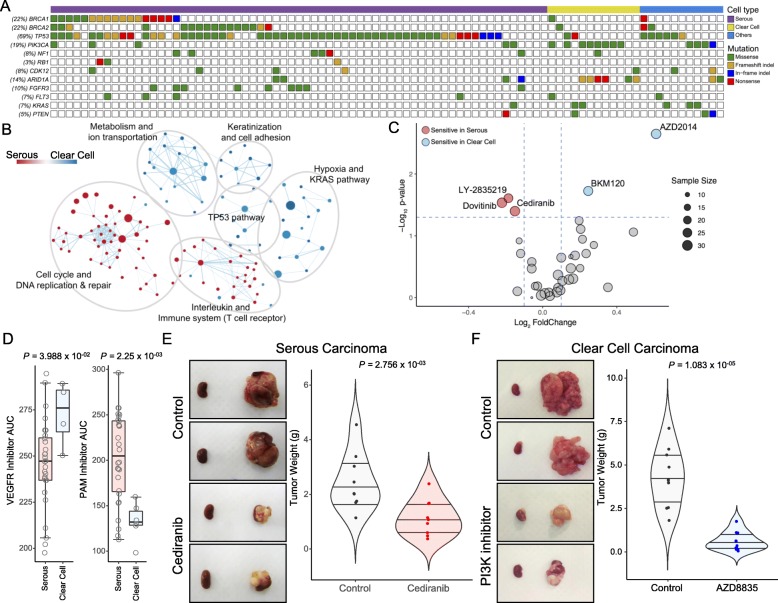
Pharmacogenomic landscape of epithelial ovarian cancer. **a** Mutational landscape of epithelial ovarian cancers. All mutations with an allele frequency of > 5% and depth of > 20 reads are shown. Genomic variations, including single nucleotide variants (SNVs), frameshift insertions/deletions, in-frame insertions/deletions, and non-sense mutations, are shown. Frequency of each genomic alteration within the whole cohort is shown on the left column. **b** Network-based enrichment map analysis of gene set enrichment results. Gene sets are organized as a network, where each gene set is a node and edges represent genes overlapping between the sets. Related gene sets are laid out as network clusters. **c** Volcano plot representation of ovarian cancer type-specific drug response, with fold-change drug comparison (*x*-axis) and its significance (*y*-axis). Each circle represents a single tumor type-drug interaction, and the size is proportional to the cohort size of respective tumor. **d** Drug response assessments of VEGFR (left panel) and PI3K-AKT-mTOR (left panel) inhibitors in serous and clear cell carcinomas. Box plots span from the first to third quartiles, and the whiskers represent the 1.5 interquartile range. **e**, **f** In vivo drug response assessments of cediranib (**e**) and PI3K inhibitor (**f**) in serous and clear cell carcinomas, respectively. Violin plots represent the overall tumor weights of the PDX models from respective groups. Horizontal lines within the violin plots represent 0.25, 0.50, and 0.75 quantiles. *P* values in **c**–**f**: two-sided Wilcoxon’s rank-sum test

EOCs can be primarily categorized into two distinct subtypes based on their histopathological features: serous and clear cell carcinomas [37]. Since histologic characteristics largely contribute to diverse molecular and phenotypic states, we suspected that a wide-range of EOC-derived PDCs would demonstrate cell type-specific pharmacogenomic interactions. We first analyzed genomic profiles of EOCs using targeted exome sequencing (Fig. 3a and Additional file 6: Table S5). The mutational frequencies of *BRCA1* and *BRCA2* were approximately 26 and 25%, respectively, in serous subtype tumors. Conversely, only a small fraction of clear cell carcinomas harbored *BRCA1* or *2* mutations (8% for both *BRCA1* and *BRCA2*; Fig. 3a). As previously reported, *TP53* mutation was highly enriched in high-grade serous carcinomas (HGSC; 82%), while only 17% of clear cell tumors harbored somatic mutation of *TP53* (*P* = 2.179 × 10^−05^) [38, 39]. Notably, clear cell carcinomas were marked by high prevalence of *PI3KCA* and *ARID1A* mutations (67% and 50% for *PIK3CA* and *ARID1A*, respectively), compared to HGSC and other EOC types (*P =* 1.067 × 10^−05^ and *P =* 5.43 × 10^−04^) [40–42]. Surprisingly, other EOCs demonstrated considerably high levels of *KRAS* and *PTEN* genomic mutations (Additional file 1: Figure S7). To explore dynamic cellular signaling pathways that are enriched between HGSC and clear cell tumors, we conducted Gene Set Enrichment Analysis (GSEA) using gene expression profiles (Fig. 3b). Interestingly, cell cycle and DNA replication/repair-associated pathways were significantly enriched in HGSC subtype. Additionally, interleukin and immune system encoding genes, including T cell receptor pathways, were profoundly more activated in HGSCs. In contrast, activations of metabolism/ion transport, keratinization/cell adhesion, hypoxia/KRAS, and TP53 signaling pathways were more predominant in clear cell tumors (Fig. 3b). Next, we examined differential drug sensitivity between HGSC and clear cell tumors to various molecularly targeted agents (Fig. 3c and Additional file 7: Table S6). Notably, several multi-kinase inhibitors, including dovitinib (FLT-3/c-Kit, FGFR1/3, and VEGFR) and cediranib (VEGFR, FIT1/4, and c-KIT), showed significantly high anti-tumor activities in HGSCs (*P* = 2.92 × 10^−02^ and *P =* 3.98 × 10^−03^ for dovitinib and cediranib, respectively). HGSCs were also widely sensitive to LY-2835219, a CDK4/6 inhibitor (*P* = 2.46 × 10^−03^), as well. As inferred by enrichment of *PIK3CA* somatic mutation, clear cell carcinomas demonstrated superior sensitivities to PAM pathway inhibitors, including BKM120 (PI3K, *P* = 1.88 × 10^−03^) and AZD2014 (mTOR, *P =* 2.25 × 10^−03^). Comparative analysis between serous and clear cell carcinomas also revealed that serous type tumors were markedly sensitive to VEGFR inhibitors, including cediranib, pazopanib, and sunitinib (Fig. 3d). These findings were consistently confirmed in vivo using PDX models that were established from HGSC (Fig. 3e) and clear cell type PDCs (Fig. 3f).

### Identification of genomic biomarkers for drug sensitivity in gynecologic cancers

**Fig. 4:**
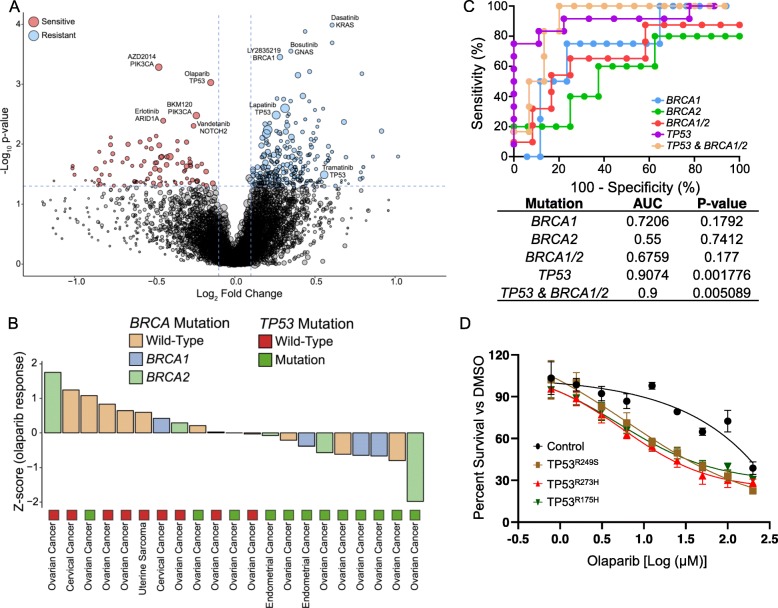
Predictive biomarkers for response to PARP inhibitors. **a** Volcano plot representation of gene-drug interactions in gynecologic tumors. **b** Waterfall plot enumerating individual tumor response to olaparib with *BRCA1/2* and *TP53* mutation status. **c** Receiver operating characteristic curve plotted for the sensitivity versus 100 - specificity values for predicting olaparib response rates using *BRCA1/2* and *TP53* mutation status. **d** Drug response assessment of olaparib on OVISE cell-line that has been stably transduced with/without *TP53*-*R249S*, *T273H*, or *R175H* mutation. Dose response curves were generated using percent survival of cells under olaparib treatment for 4 days on 9 different doses from 200 to 0.78 μM. *P* values in **a**—two-sided Wilcoxon’s rank-sum test, and in **c**—two-sided binomial exact test

Previous studies have shown that a single genetic alteration could be employed as a surrogate biomarker for predicting clinical response to various drug classes [4, 5, 9, 10, 12, 13, 19]. To identify genomic correlates of diverse pharmacological responses in gynecologic tumors, we evaluated individual drug profiles against single genomic alterations (Fig. 4a, Additional file 8: Table S7). Among the numerous interactions, *PIK3CA* mutation was the most robust genomic predictor of sensitivity towards PAM pathway inhibitors, including AZD2014 (mTOR, *P* = 5.20 × 10^−04^) and BKM120 (PI3K, *P* = 3.34 × 10^−03^). Pharmacological activities of erlotinib (EGFR) and vandetanib (VEGFR) were significantly associated with genomic aberrations of *ARID1A* (*P* = 4.07 × 10^−03^) and *NOTCH2* (*P* = 4.96 × 10^−03^), respectively. *BRCA1* mutation was also linked to therapeutic resistance against a CDK4/6 inhibitor, LY2835219 (*P =* 3.53 × 10^−04^). Gynecologic PDCs that harbored somatic mutations in *KRAS* and *GNAS* were widely resistant to dasatinib (Bcr-Abl, *P* = 1.03 × 10^−04^) and bosutinib (Abl and SRC, *P* = 2.81 × 10^−04^), respectively. *TP53-*mutant tumors were highly resistant to a wide range of therapeutics, including lapatinib (EGFR, *P* = 2.52 × 10^−03^), AZD5363 (AKT, *P* = 3.28 × 10^−03^), and trametinib (MEK, *P* = 3.24 × 10^−02^). Conversely, *TP53* mutated tumors were significantly sensitive to olaparib (PARP) treatment (*P* = 9.39 × 10^−04^).

PARP inhibition demonstrated potent therapeutic efficacies in patients who were diagnosed with either metastatic breast or advanced ovarian cancers with germline BRCA1/2 mutations [43, 44]. Although statistically not significant, BRCA1/2 mutations were enriched in olaparib-sensitive PDCs (represented with *Z*-score < 0) (Fig. 4b). Interestingly, *TP53* mutation also portrayed profound anti-tumor activity towards olaparib, regardless of histopathological subtype. Receiver operating characteristic (ROC) analysis revealed that genomic alterations of BRCA1/2 demonstrated positive correlations with olaparib sensitivity (AUC of ROC > 0.5; Fig. 4c). Furthermore, sole *TP53* mutation or combination of *TP53* with BRCA1/2 mutations revealed enhanced predictability to olaparib treatment (AUC = 0.9074 and 0.9 for *TP53* and *TP53* with *BRCA1/2*, respectively; Fig. 4c). To functionally validate our findings, we established cancer cell-line models that stably express various dominant-negative mutant forms of *TP53* (R273H, R249S, and R175H) [45] in an OVISE ovarian cancer cell-line (*TP53* wild-type). As suspected, cytotoxic activities of olaparib were significantly enhanced on all *TP53* mutants (log (IC_50_) values for *TP53* wild-type, R273H, R249S, and R175H mutants were 2.155 (95% CI 2.055 to 2.259), 1.421 (95% CI 1.336 to 1.506), 1.269 (95% CI 1.177 to 1.362), and 1.408 (95% CI 1.296 to 1.520) μM, respectively) (Fig. 4d).

### Transcriptomic correlates of olaparib response in EOCs

**Fig. 5:**
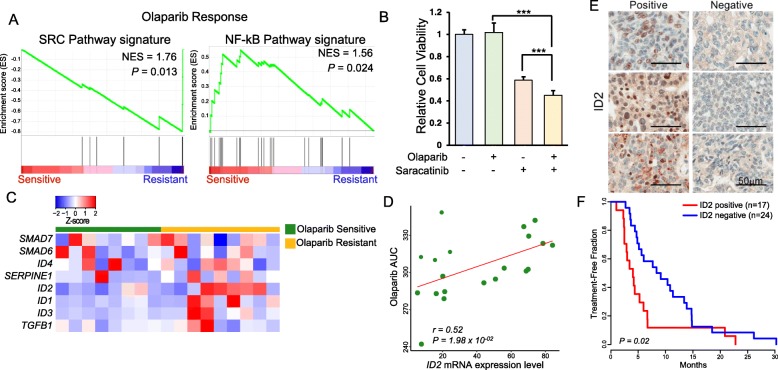
Transcriptomic correlates of olaparib sensitivity. **a** Gene Set Enrichment Analysis (GSEA) between olaparib-sensitive and olaparib-resistant PDCs. **b** Drug response assessment of olaparib and/or saracatinib. **c** Heatmap representation of SRC pathway encoding gene expressions in olaparib-sensitive and olaparib-resistant PDCs. **d** A scatter plot demonstrating linear correlation between olaparib AUC and *ID2* expression. The correlation coefficient and the *P* value were obtained using Pearson’s correlation test. **e** Representative immunohistochemical images of ID2 staining in patient tumor specimens. Scale bars, 50 μm. **f** The Kaplan-Meier treatment-free survival analysis of patients with high vs. low ID2 protein expression levels. *P* values in **b**—two-tailed *t* test, in **d**—Pearson’s correlation test, and in **f**—log-rank test

Transcriptome analysis enables identification of unique gene-signature correlates for particular drug sensitivity [12, 13, 19]. To identify potential molecular determinants to olaparib response, we systematically analyzed the transcriptome profiles of EOCs based on their pharmacological responses to olaparib [44]. We discovered that expressions of SRC pathway encoding genes were significantly enriched in olaparib-resistant PDCs, while olaparib-sensitive tumors were marked by activation of NF-kB pathway (Fig. 5a). Functional validation of SRC inhibition showed that saracatinib (SRC, 20 μm) significantly augmented the therapeutic effects of olaparib in *BRCA1*-mutant PDCs (*P =* 3.73 × 10^−04^; Fig. 5b). Among the list of enriched SRC pathway-encoding genes, mRNA expressions of ID1/2/3 were predominantly upregulated in olaparib-resistant PDCs (Fig. 5c). Especially, transcriptome expression level of *ID2* was significantly correlated with therapeutic resistance to olaparib treatment (*r* = 0.52, *P* = 0.01978; Fig. 5d). To determine whether *ID2* expression could be employed as a potential predictor of response to olaparib, we retrospectively analyzed treatment-free survival of 41 patients (ID2 positive, *n* = 17; ID2 negative, *n* = 24), who were previously diagnosed with HGSC, harbored *BRCA1* or *2* mutation, and received olaparib treatment. Notably, the Kaplan-Meier survival analysis revealed that patients with low ID2 expression demonstrated significantly prolonged treatment-free survival to olaparib (log rank test, *P = 0.02*, median survival: ID2^pos^ 4.03 months vs. ID2^neg^ 8.73 months; 95% CI: ID2^pos^ 2.87~6.67 vs. ID2^neg^ 5.60~13.87; Fig. 5e, f). Collectively, our results indicate that enrichments of SRC pathway and *ID2* expression relate to therapeutic resistance to olaparib treatment in EOCs.

## Discussion

The success of precision oncology depends on identification of effective drugs tailored to individual patients based on molecular profiling of the tumor. Comprehensive analyses of cancer genome have revealed a landscape of key genetic ablations that constitute complex process of tumorigenesis [1, 8, 46]. A large-scale compilation of pharmacological drug response with molecular characterization of cancer cell-line models has provided a reference point for evaluating potential genomic markers of drug sensitivity [12, 13, 16, 17]. Moreover, we have previously established a landscape of pharmacogenomic interactions using a library of short-term cultured PDCs to explore dynamic gene-drug associations and presented its clinical feasibility [19]. As an extension, we generated a collection of 139 gynecologic tumors from patients with cervical, endometrial/uterine, or ovarian cancers. Through integrative genomic, transcriptomic, and pharmacological analyses, we have provided several new therapeutic insights for gynecologic malignancies (Fig. 1a).

We evaluated lineage-specific drug sensitivity in gynecologic tumors and discovered that EOCs demonstrated enhanced sensitivities to multiple EGFR inhibitors, while ECs were particularly sensitive to everolimus, an mTOR inhibitor. A number of clinical observations further advocated our results. Despite the small number of patients and lack of randomization, addition of erlotinib (EGFR) to platinum or paclitaxel provided favorable clinical outcomes for EOC patients, compared to platinum or paclitaxel treatment alone [47]. Moreover, several clinical investigations demonstrated potential therapeutic benefits of mTOR targeted therapy in EC patients [36, 48–50]. Consistent with previous observations, we confirmed that serous and clear cell EOCs can be distinguished by evidently distinct genomic compositions (Fig. 3a), highlighting the need for molecular-based therapeutic approaches. Interestingly, our drug screening results, coupled with in vivo validations, propose clinical utility of cediranib (VEGFR) for HGSC patients, while PAM inhibitors could be more beneficial for those with clear cell carcinomas. Recent phase II and III clinical trials with cediranib also revealed that cediranib plus olaparib treatment resulted in a significant improvement in progression-free survival of HGSC patients compared to olaparib alone [51, 52]. As in vivo PDX results were only representative cases, further functional validations in a larger cohort are warranted to explore potential clinical applications of VEGFR and PAM inhibitors in serous and clear cell type tumors, respectively.

We also identified genomic correlates of drug sensitivity and resistance to olaparib. Approximately 13–18% of the HGSCs are attributable to *BRCA1* or *2* germline mutations, and PARP inhibition therapy has been a successful approach for these patients. However, the need for discovering an alternative molecular biomarker to better predict the clinical efficacy against PARP inhibition treatment has been increasingly recognized due to global acquisition of olaparib resistance. Notably, we demonstrated that addition of *TP53* mutation could be a significant molecular determinant for predicting potential clinical response to PARP inhibition therapy. The tumor suppressor protein p53 provides an essential role in governing cell cycle arrest or apoptosis upon DNA damage [53]. However, the underlying molecular cascades that determine p53 protein stability and its activation are not fully understood. Accumulation of PARP1 is an early event where a single strand DNA break is generated and initiates base excision repair (BER) pathway [54]. PARP-1 interacts with p53 to modulate DNA damage [55], and genotoxic drugs promote accumulation and activation of p53 in parp-1-deficient cells. Furthermore, p53 deficiency enhanced pharmacological sensitivity towards PARP inhibition therapy in mantle cell lymphoma [55]. Consistent with such findings, our results suggest the prospect of targeting p53-deficient tumors with PARP inhibitors regardless of histopathological characteristics. Prevalence of *TP53* mutation in HGSCs could contribute to the clinical success of PARP inhibitors [39, 42, 44].

Lastly, we identified SRC activation to be associated with therapeutic resistance to olaparib. The SRC family of non-receptor tyrosine kinases regulates essential cellular programs, including cellular proliferation, differentiation, migration, survival, and angiogenesis [56]. A substantial number of studies have postulated that activation of SRC pathway contributes to inherent resistance to chemotherapy and inhibition of SRC pathway could potentially circumvent such mechanism [57, 58]. Moreover, transcriptional expressions of *ID* family genes were identified as molecular correlates of olaparib sensitivity. ID proteins are members of the large family of the helix-loop-helix (HLH) transcription factors. During development, ID proteins govern cell cycle and differentiation programs by modulating various cell cycle regulators [59]. From the perspective of tumorigenesis, upregulation of ID protein is mediated by a group of proto-oncogenes, including *Myc*, *Ras*, and (*EWS*)*-Ets*, and prevents activation of various tumor suppressor genes [60], making it a promising therapeutic target [61].

## Conclusion

Here, we generated an additional cohort at single tumor-lineage resolution, specializing in gynecologic malignancies. We performed systematic analyses of tumor genome and transcriptome to identify molecular determinants that dictate drug sensitivity to various molecular targeted drugs that are currently being used or under development. Our work provides an extension to current pharmacogenomic database in identifying predictive biomarkers and combinational approach to overcome cellular-intrinsic resistance to particular drug classes, including PARP inhibitors.

## Methods

### Gynecologic cancer specimens and their derivative cells

After receiving informed consent, tumor specimens and clinical records were obtained from patients undergoing surgery at Samsung Medical Center (SMC) in accordance with its Institutional Review Board. Surgical samples measuring ~ 5 × 5 × 5 mm^3^ were snap frozen using liquid nitrogen for genomic analysis. Portions of the surgical samples were enzymatically dissociated using Liberase TM (Roche) and cultured in DMEM/F12 media with l-glutamine (Thermofisher), N2 and B27 supplements (0.5× each; Thermofisher), human recombinant basic fibroblast growth factor (bFGF), and epidermal growth factor (EGF; 20 ng/ml each; R&D Systems).

### Orthotopic xenograft animal models and drug treatment

Female BALB/c nude mice were purchased from ORIENT BIO (Sungnam, Korea). This study was performed in accordance with relevant guidelines and regulations. This study was reviewed and approved by the Institutional Animal Care and Use Committee (IACUC) of Samsung Biomedical Research Institute (SBRI). SBRI is an Association for Assessment and Accreditation of Laboratory Animal Care International (AAALAC International, protocol no. H-A9-003)-accredited facility and abides by the Institute of Laboratory Animal Resources (ILAR) guidelines. To generate PDX models, patient tumor specimens were cut into small pieces (below 2–3 mm), implanted into the subrenal capsule of the left mouse kidney, and propagated by serial transplantation [34]. After 1–2 weeks, the mice (*n* = 10 mice per group) were treated with either 1% polysorbate 80 or 0.5% methylcellulose or cediranib (6 mg/kg, qd, p.o.) or AZD8835 (25 mg/kg, bid, p.o.). Mice were monitored daily for tumor development and postoperative complications and were sacrificed between day 35 and 40 or if mice seemed moribund. Total body weight and tumor weight of each mouse were recorded. Tumors were fixed in formalin and embedded in paraffin or snap frozen in the OCT compound (Sakura Finetek, Japan, Tokyo, Japan) in liquid nitrogen.

### Isolation of genomic DNA and quality control

Genomic DNA was extracted from fresh tissue specimens using the QIAamp DNA mini kit (Qiagen, Valencia, CA, USA) or from FFPE tissues using either the Promega Maxwell 16 CSC DNA FFPE kit or the QIAamp DNA FFPE Tissue kit according to the manufacturer’s manual. The purity, amount, and median size of the extracted DNA were measured by the Nanodrop 8000 UV-Vis spectrometer (Thermo Scientific Inc., Wilmington, DE, USA), Qubit 2.0 fluorometer (Life Technologies Inc., Grand Island, NY, USA), and 2200 TapeStation Instrument (Agilent Technologies, Santa Clara, CA, USA). In addition, ΔCt values were determined using real-time PCR (Agilent Technologies) with Mx3005p instrument (Agilent Technologies, USA), FFPE QC kit (Illumina, cat no. WG-321-1001), and Brilliant Ultra-Fast SYBR Green qPCR (Agilent Technologies, cat no. 600882). If DNA meets the quality criteria such as (i) purity to absorption ratio (260 nm/280 nm) > 1.8, 260 nm/230 nm > 1.8; (ii) total amount > 250 ng; (iii) degradation to ΔCt value < 2.0; or DNA median size > 0.35 kb, it is proceeded onto the sequencing step.

### Panel design and sequencing

Samples were profiled on CancerSCAN, a targeted sequencing platform designed at Samsung Medical Center. This customized platform offers flexibility to include target genes that are curated from the literature or requested by researchers and clinicians. To obtain cancer panel sequencing data, CancerSCAN probes were designed to enrich the exons of 80 target genes (Additional file 9: Table S8). Genomic DNA was sheared using the Covaris S220 (Covaris, Woburn, MA) to construct a sequencing library using the SureSelect XT Reagent Kit, HSQ (Agilent Technologies) on target genes. A paired-end sequencing library was purified and amplified with a barcode tag, and the library quality and quantity were determined. Sequencing was carried out using the 100-bp paired-end mode of the TruSeq Rapid PE Cluster kit and TruSeq Rapid SBS kit on a HiSeq 2500 sequencing platform (Illumina, San Diego, CA, USA).

### Bulk RNA sequencing

RNA-seq libraries were prepared using the Illumina TrueSeq RNA Sample Prep kit. Sequenced reads were mapped onto hg19 using the Burrows-Wheeler Aligner (BWA). The initial alignment BAM files were sorted and summarized into BED files using SAMtools and bedTools. The BED files were used to calculate values of RPKM (reads per kilobase of transcript per million reads) for each gene, using DEGseq package.

### Drug screening

PDCs were cultured in serum-free medium, dissociated into single cells, and seeded in 384-well plates at a density of 500 cells per well in duplicate or triplicate for each treatment. The drug panel consisted of 37 anti-cancer agents targeting oncogenic signals. All drug libraries were purchased from Selleckchem. PDCs were treated with drugs in a four-fold and seven-point serial dilution series from 20 to 4.88 nM using a Janus Automated Workstation (PerkinElmer, Waltham, MA, USA). After 7 days of incubation at 37 °C in a 5% CO_2_ humidified incubator, cell viability was analyzed using an adenosine triphosphate (ATP) monitoring system based on firefly luciferase (ATPLite™ 1step, PerkinElmer). Viable cells were estimated using an EnVision Multilabel Reader (PerkinElmer). The controls, dimethyl sulfoxide (DMSO) vehicle, were used to calculate relative cell viability for each plate and to normalize the data on a per-plate basis. Dose response curve (DRC) fitting was performed using GraphPad Prism 5 (GraphPad) and evaluated by measuring the area under curve (AUC). In brief, each plate was normalized to the mean of the seven conditions on the plate with a DMSO control. After normalization, best-fit lines and the resulting IC_50_values were calculated using GraphPad: [log(inhibitor) vs. response − variable slope (four parameters)]. *Y* = Bottom + (Top − Bottom)/(1 + 10^((logIC_50_ − *X*) × HillSlope)). The AUC of each curve was determined using GraphPad Prism, ignoring regions defined by fewer than two peaks. Non-convergence or ambiguous curves are excluded in every analysis.

### Pharmacogenomic interactions on major genomic alterations

For gene-drug associations, a list of major cancer-driver alterations, including single nucleotide variations, small insertions, and deletions, was considered as a predictive genomic biomarker to evaluate drug response interactions. For each drug candidate, drug sensitivity data (AUCs) were analyzed by comparing tumors with the selected genomic alteration to those without using the Wilcoxon rank-sum test. Samples with unknown status of a given alteration were excluded from the analysis. To evaluate lineage-specific drug sensitivities in gynecologic tumors, drug sensitivity data were analyzed by comparing tumors from each pathologic entity to the rest using the Wilcoxon rank-sum test. For transcriptome analysis, tumors were classified as “sensitive” (*Z*-score < − 0.5) or “resistant” (*Z*-score > 0.5) based on drug sensitivity data and subjected to Gene Set Enrichment Analysis (GSEA).

### Plasmid preparation and stable cell establishment

Lentiviral vectors encoding TP53 mutants (R175H, R273H, or R249S) in pLenti6/V5 plasmid were purchased from Addgene (cat no. 22936, 22934, and 22935, respectively). Lentivirus was prepared by transfecting plasmids into the 293T cells using pMD2G, psPAX2 (Addgene), and Lipofectamine 2000 (Thermofisher). After the initial transfection, supernatants were collected after 48 and 72 h and filtered through 0.45 μM filters (Milipore). To generate stable TP53 mutant-expressing cell lines, lentivirus particles were incubated with ovarian clear cell carcinoma, OVISE, and treated with polybrene (8 μg/ml, Sigma) for 48 h and blasticidin (5 μg/ml, Sigma) selection was performed for 2 weeks.

### Immunohistochemistry

Immunohistochemical staining was performed on formalin-fixed, paraffin-embedded, 4–5-μm-thick tissue sections, using the Bond-maxTM automated immunostainer (Leica Biosystems, Melbourne, Australia) and BondTM Polymer Refines Detection Kit (Vision Biosystems, Melbourne, Australia). Mouse monoclonal anti-ID2 antibody (1:100; NBP2-66898, Novus Biologicals, USA) was used. Briefly, antigen retrieval was carried out at 97 °C for 20 min in ER1 buffer. After blocking the endogenous peroxidase activity with 3% hydrogen peroxidase for 10 min, primary antibody was incubated for 60 min at room temperature. To verify antibody specificity, anti-mouse IgG (AI-2000; Vector Laboratories, Burlingame, CA, USA) was used as a control. The degree of immunostaining of ID2 was evaluated according to staining proportion of positively stained cancer cell nucleus and the staining intensity, as previously described [62]. Briefly, the areas of stained cancer cells were scored as follows: the percentage of positive cells (0–100%) and intensity scaled from 0 to 2 (null = 0, weak to moderate = 1, strong = 2).

### Statistics

All statistical analyses were conducted by either Wilcoxon’s rank-sum test (two-sided), Pearson’s correlation coefficient test, or Fisher’s exact test (two-sided). Survival curves were estimated with the Kaplan-Meier method. All statistical analyses were conducted and obtained using the R software (https://www.r-project.org).

## Supplementary information

**Additional file 1: Figure S1**. Frequency of major cancer-driver gene alterations in gynecologic tumors. **Figure S2**. Frequency of major cancer-driver genes in SMC and TCGA cohorts. **Figure S3**. Drug panel classification. **Figure S4**. Preservation of copy number alterations in primary tissues and PDCs. **Figure S5**. Representation of histopathologic characteristics between parental and patient-derived xenograft tumors. **Figure S6**. Pharmacological landscape of 37 molecular-targeted agents. **Figure S7**. Frequency of major cancer-driver gene alterations in ovarian cancer.

**Additional file 2: Table S1.** Descriptive characteristics of 139 samples from gynecologic cancers used in the drug screening and/or the genomic evaluations.

**Additional file 3: Table S2.** A list of the 37 drugs used in drug sensitivity analysis. The drugs were described by the chemical and/or generic names, their respective targets and clinical phases.

**Additional file 4: Table S3.** Area under the curve (AUC) values of 37 drugs in 66 PDCs. NA represents the AUC value extracted from non-fitted dose response curve (DRC) that resulted in a non-convergent or ambiguous curve.

**Additional file 5: Table S4.** Tumor type-specific drug associations identified using 37-drug library. Wilcoxon rank sum test was applied to determine the relative differences of drug sensitivity between certain tumor type and all the other cancers.

**Additional file 6: Table S5.** The genomic profile of gynecologic tumor samples which was identified using CancerSCANTM sequencing and analysis protocol.

**Additional file 7: Table S6.** Cell type-specific drug associations in EOCs. Wilcox rank sum test was applied to determine the relative differences of drug sensitivity between serous and clear cell type tumors.

**Additional file 8: Table S7.** Pharmacogenomic associations identified using integrative analysis of drug sensitivity results (AUC) and genomic alteration. The statistical significance was calculated using Wilcoxon rank sum test.

**Additional file 9: Table S8.** List of genes (*n* = 80) and sequencing protocol for CancerSCANTM, detecting cancer-driven variant.

**Additional file 10.** Review History.

## Data Availability

All sequenced data have been deposited in the European Genome-phenome Archive (EGA) under accession EGAS00001003556 [63].
