# Supplementary material for: Pharmacogenomic analysis of patient-derived tumor cells in gynecologic cancers
Source: Genome Biol. 2019 Nov 26;20:253. doi: 10.1186/s13059-019-1848-3 (PMC6880425; doi:10.1186/s13059-019-1848-3)
Supplement: Supplementary file 1 — Additional file 1: Figure S1. Frequency of major cancer-driver gene alterations in gynecologic tumors. Figure S2. Frequency of major cancer-driver genes in SMC and TCGA cohorts. Figure S3. Drug panel classification. Figure S4. Preservation of copy number alterations in primary tissues and PDCs. Figure S5. Representation of histopathologic characteristics between parental and patient-derived xenograft tumors. Figure S6. Pharmacological landscape of 37 molecular-targeted agents. Figure S7. Frequency of major cancer-driver gene alterations in ovarian cancer. [file 13059_2019_1848_MOESM1_ESM.pdf]

**Supplementary Information**

# Pharmacogenomic analysis of patient-derived tumor cells in gynecologic cancers

Jason K. Sa, Jae Ryoung Hwang, Young-Jae Cho, *et al*

## 1. Supplementary Figures and legends

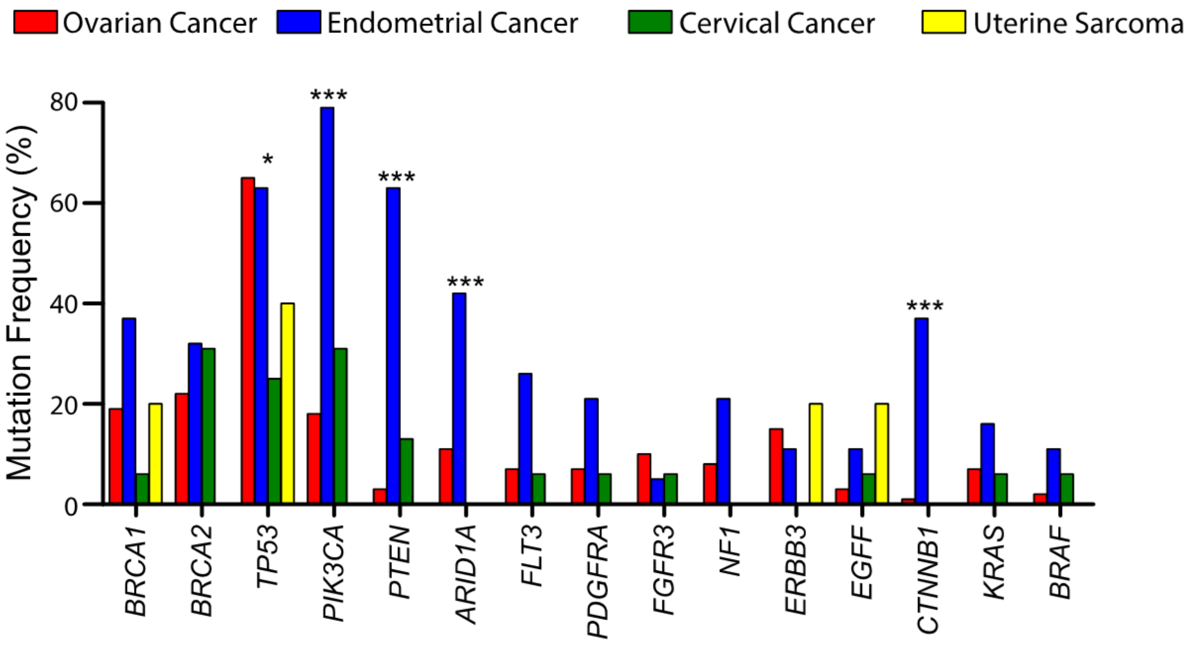


**Figure S1. Frequency of major cancer-driver gene alterations in gynecologic tumors**

Frequency of major cancer-driver alterations are denoted based on histopathological classifications: Ovarian cancer, Endometrial cancer, Cervical cancer, and Uterine Sarcoma.

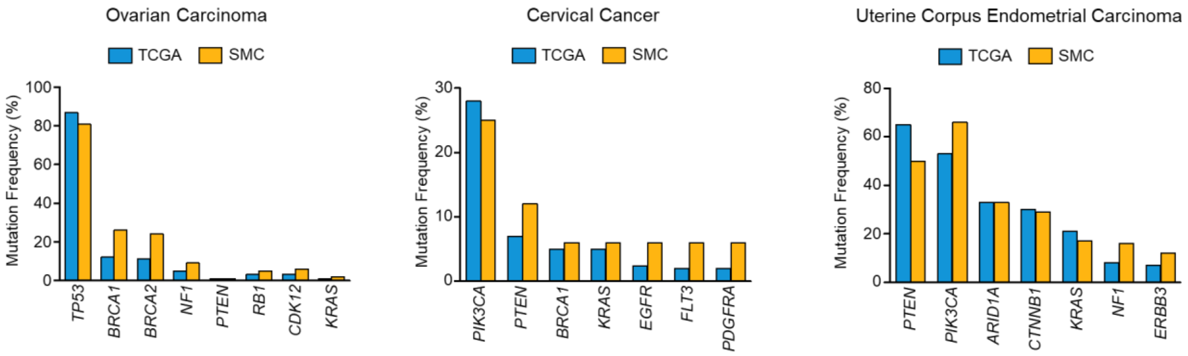


**Figure S2. Frequency of major cancer-driver genes in SMC and TCGA cohorts**

Frequency of major cancer-driver mutations are evaluated between SMC and TCGA cohorts in ovarian carcinoma, cervical cancer and uterine corpus endometrial carcinoma

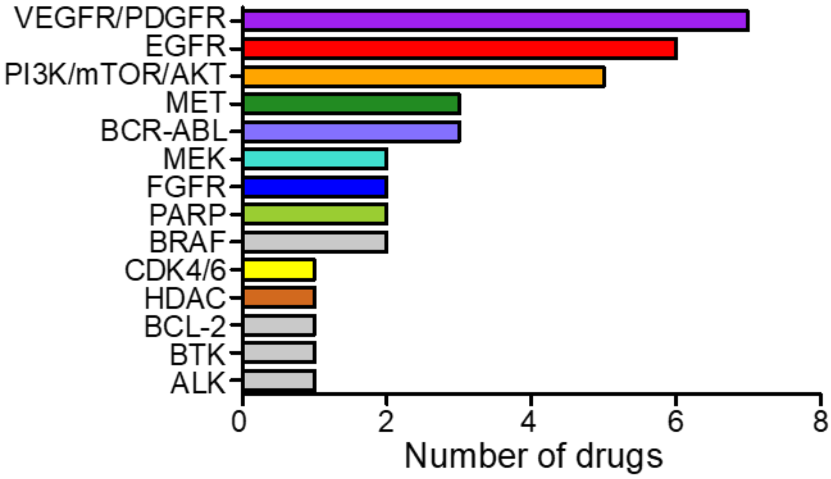


**Figure S3. Drug panel classification**

The panel of 37 drugs are classified according to their therapeutic targets.

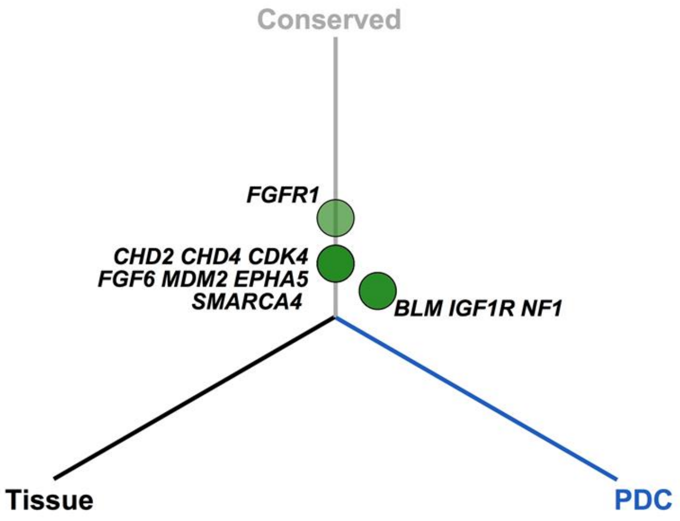


**Figure S4. Preservation of copy number alterations in primary tissues and PDCs**

Three-dimensional bubble plot demonstrating the frequency of cancer-driver copy number alterations exclusively in tissue (black, left axis), in PDC (blue, right axis), or shared between the two (grey, upper axis).

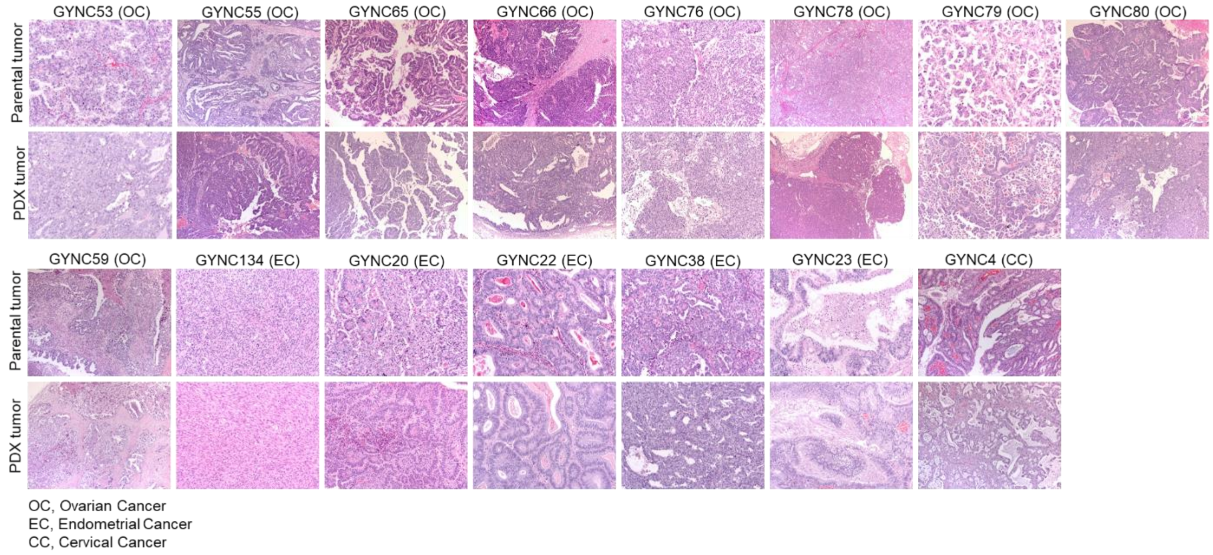


**Figure S5. Representation of histopathologic characteristics between parental and patient-derived xenograft tumors**

Representative immunohistochemical images of patient tumors with corresponding xenograft tumors in ovarian, endometrial and cervical caners

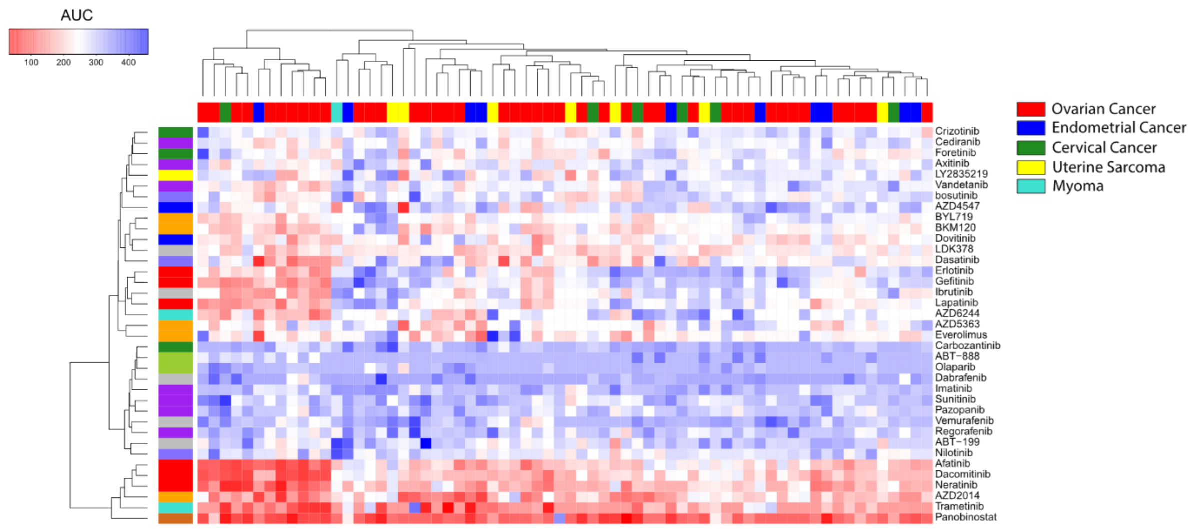


**Figure S6. Pharmacological landscape of 37 molecular-targeted agents**

Hierarchical clustering of AUC-based drug sensitivities, annotated by their origin of tissues. The red color on the heatmap represents sensitivity, while the blue color denotes resistance. Drugs were clustered based on their general responses.

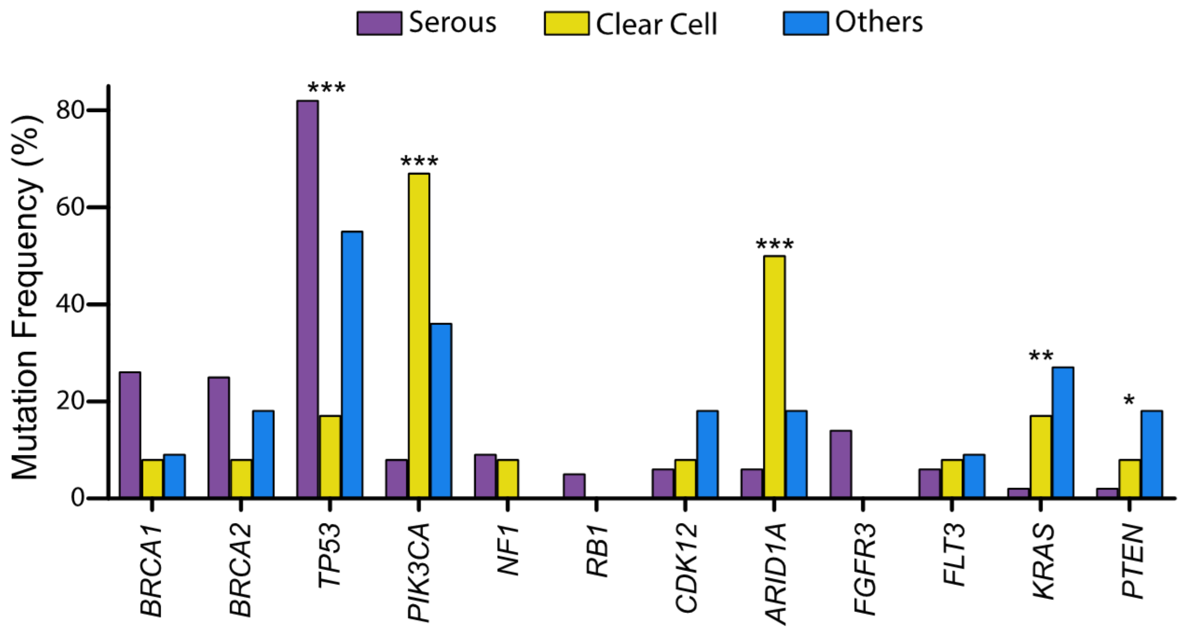


**Figure S7. Frequency of major cancer-driver gene alterations in ovarian cancer**

Frequency of major cancer-driver alterations in ovarian cancer based on their histopathological subtypes: serous carcinoma, clear cell carcinoma, and others.

## 2. Supplementary table legends

**Table S1.** Descriptive characteristics of 139 samples from gynecologic cancers used in the drug screening and/or the genomic evaluations

**Table S2.** A list of the 37 drugs used in drug sensitivity analysis. The drugs were described by the chemical and/or generic names, their respective targets and clinical phases.

**Table S3.** Area under the curve (AUC) values of 37 drugs in 66 PDCs. NA represents the AUC value extracted from non-fitted dose response curve (DRC) that resulted in a non-convergent or ambiguous curve.

**Table S4.** Tumor type-specific drug associations identified using 37-drug library. Wilcoxon rank sum test was applied to determine the relative differences of drug sensitivity between certain tumor type and all the other cancers.

**Table S5.** The genomic profile of gynecologic tumor samples which was identified using CancerSCAN$^{TM}$ sequencing and analysis protocol.

**Table S6.** Cell type-specific drug associations in EOCs. Wilcox rank sum test was applied to determine the relative differences of drug sensitivity between serous and clear cell type tumors.

**Table S7.** Pharmacogenomic associations identified using integrative analysis of drug sensitivity results (AUC) and genomic alteration. The statistical significance was calculated using Wilcoxon rank sum test.

**Table S8.** List of genes (n = 80) and sequencing protocol for CancerSCAN$^{TM}$, detecting cancer-driven variant.
