# Supplementary material for: Pharmacogenomic analysis of patient-derived tumor cells in gynecologic cancers
Source: Genome Biol. 2019 Nov 26;20:253. doi: 10.1186/s13059-019-1848-3 (PMC6880425; doi:10.1186/s13059-019-1848-3)
Supplement: Supplementary file 10 — Additional file 10. Review History. [file 13059_2019_1848_MOESM10_ESM.docx]

Review History

**First round of review**

**Reviewer 1**

**Are you able to assess all statistics in the manuscript, including the appropriateness of statistical tests used?**

yes

**Comments to author:**

In this manuscript, Sa et al established a library of 139 gynecologic tumor genomically and/or pharmacologically annotated and explored dynamic pharmacogenomic associations against molecularly targeted drugs. They discovered lineage-specific drug sensitivities based on subcategorization of gynecologic tumors and identified TP53 mutation as a molecular determinant that elicits therapeutic response to PARP inhibitors. The authors further identified transcriptome expression of ID2 as a potential predictive biomarker for treatment response to olaparib. Overall, this study is novel, interesting and of clinical significance. However, I still have several concerns listed below.

1.     Although it is generally ok to perform exome sequencing and/or RNA seq for both PDCs and parental tumors to demonstrate their molecular similarity, have you also examined the tumorigeneity or these PDCs? If so, what about the histological assessment of those PDX models established with those PDCs compared with their parental tumors? Are they still histologically similar?
2.     For Figure 3e and Figure 3f, have you checked the impact of each drug on the survival of each subtype gynecological cancers?
3.     To enable reproducibility by other research groups, it is recommended to provide the detailed algorithms to calculate the gene-drug associations used in this study.
4.     In the initial study that incorporated 139 gynecological tumor specimens, it seems that the number of each tumor type is quite unbalanced, with 18 cervical cancer samples, 29 uterine/endometrial cancer samples, and 92 epithelial ovarian cancer samples included. How could you manage to guarantee an unbiased analysis among the three types of gynecological cancers with an unbalanced number of tumor sampels included? How can you ensure the representativeness of these limited number of samples for each cancer type, especially for cervical cancer and uterine/endometrial cancer?
5.     For figure 5f, the number of ID2 positive patients is too small (n=3). Please enlarge the sample size for analysis.
6.     Please invite a native speaker to polish the language of the entire manuscript.

**Reviewer 2**

**Are you able to assess all statistics in the manuscript, including the appropriateness of statistical tests used?**

yes

**Comments to author:**

In this manuscript, the authors established a library of 139 gynecologic tumors, as well as 66 PDCs. The experimental protocol is largely followed their previous publications, but with different emphasize of cancer types for gynecologic cancers. The pharmacogenomics data exhibits as a good resource, but the analysis and experiments need further improvement. 

Major concern:
1. It is critical to establish the link between tumor biospecimen and PDCs, as the authors did in their Nature Genetics paper. However, in this manuscript, the evidence is not strong. For example, authors showed the mutational landscape in PDCs, but several PDCs do not have any key mutations at all (Figure 1b). For each key mutation gene, do they have similar mutation frequency in PDC compared to tumor samples? It is unclear how the authors make the comparison in figure 1 c (upper panel). In figure 1c (bottom panel), transcriptomic expression, it is unclear how many samples are involved in this spearman correlation? If only 6 samples are included, why only selected 6 samples? How many genes do they select for this spearman correlation? Furthermore, for ovarian cancer, CNV may be the dominant feature, and the authors may also show the similarity of CNV between PDC and tumor samples.

2. TCGA consortium has performed a comprehensive analysis across gynecologic tumors, which covered >2500 samples (Berger et al., Cancer Cell, 2018). The authors should also compare the genomic features of their tissues samples and PDCs with TCGA data.

3. The authors collected 139 tumor, but only select 66 PDCs for the follow up study? Any reason for this? Most of the PDCs are EOCs, Any reason for this? Are EOCs easier to set up PDCs? E.g., EOCs are easier to culture in EGF medium than any other gynecological tumors?

4. In figure 2c, the authors should make a pair-wise comparisons among all cancer types.

5. It is unclear for the PDX model validation. For example, the authors compared serous and clear cell in PDC model (figure 3d), while they compared serous/clear cell and control in PDX model (figure 3e/f). My understanding is that the authors want to compare the serous and clear cell, so it is necessary to add clear cell PDX in figure 3e, and add serous carcinoma in figure 3f.

6. In figure 4, the author provide evidence to prove that tumors have TP53 mutation would be more sensitive to olaparib, but the authors may need more evidence. For example, the authors showed only 20-30% reduction on cell viability introduced by BRCA1 and/or TP53 mutants, compared to at least 60% reduction in their NG paper. Furthermore, the authors may need additional cell line to confirm this result. In the discussion, the authors mentioned: "Notably, we demonstrated that addition of TP53 mutation could be a significant molecular determinant for PARP inhibition therapy". Again, the evidence is not strong enough. Importantly, the dosage is quite high, but still not strong effect. Any reason for this? The authors may consider to try different dosages.

7. For figure 5d, it is unclear the correlation is Spearman correlation or Pearson correlation.
8. Figure 5f, the sample size is too small for this kind of analysis, especially for ID2 positive samples (n=3).

Minor concern:
1. Multiple references are not correctly cited. For example, the authors sated "We have established a library of .." and cite [26]. However, the reference author list is not the current authors, and the paper is not the paper the authors want to mention. The authors need to correct their reference list to provide clear background.
2. The p-value format is not correct throughout the manuscript.
3. There are multiple grammar errors and typos need to be addressed.
4. Figure 1c lacks the x and y axis label.
5. Figure 5a lacks y axis label for enrichment score.
6. The y axis of fig 4b is missing.

**Authors Response**

**Point-by-point responses to the reviewers’ comments:**

Response to Reviewer #1 

General Remark: “In this manuscript, Sa et al established a library of 139 gynecologic tumor genomically and/or pharmacologically annotated and explored dynamic pharmacogenomic associations against molecularly targeted drugs. They discovered lineage-specific drug sensitivities based on subcategorization of gynecologic tumors and identified TP53 mutation as a molecular determinant that elicits therapeutic response to PARP inhibitors. The authors further identified transcriptome expression of ID2 as a potential predictive biomarker for treatment response to olaparib. Overall, this study is novel, interesting and of clinical significance. However, I still have several concerns listed below.” 

*Response: We thank the Reviewer for summarizing our work and providing positive assessment of our results. We especially thank the reviewer for providing constructive suggestions to improve our manuscript.* 

Major Point 1: Although it is generally ok to perform exome sequencing and/or RNA seq for both PDCs and parental tumors to demonstrate their molecular similarity, have you also examined the tumorigeneity or these PDCs? If so, what about the histological assessment of those PDX models established with those PDCs compared with their parental tumors? Are they still histologically similar? 

*Response 1: We thank the reviewer’s suggestion. Using patient-derived xenograft (PDX) models that were propagated using our PDCs, we evaluated histological features between PDX models with their parental tumors. Notably, PDX models recapitulated the original morphologic and pathologic characteristics of their parental tumors, including ovarian, endometrial and cervical cancers. Also, we added descriptions regarding these observations (page 6, highlighted in yellow and additional file 1: Fig. S5)* 

Major Point 2: For Figure 3e and Figure 3f, have you checked the impact of each drug on the survival of each subtype gynecological cancers? 

*Response 2: We appreciate the reviewer’s comment. Unfortunately, we haven’t checked the impact of each drug on the survival of our PDX models. However, a previous study validated the clinical predictive value of measuring tumor weight/volume in EOCs PDX model: The response to paclitaxel-carboplatin chemotherapy and EGFR targeted treatment in PDXs were concordant with the clinical response of the corresponding patient [1]. In such context, we suspect that suppression of both tumor weight that were observed in our results could potentially predict the clinical response and survival. Furthermore, please note that evaluating survival period of tumor bearing mice with subrenal injection would raise ethical problems: NIH Animal Research Advisory Committee Guidelines strongly recommend that mice harboring tumors over 20 mm in any dimension should be sacrificed before tumor-associated death (https://oacu.oir.nih.gov/animal-research-advisory-committee-guidelines).* 

Major Point 3: To enable reproducibility by other research groups, it is recommended to provide the detailed algorithms to calculate the gene-drug associations used in this study. 

*Response 3: We apologize for not being explicit in providing detailed algorithms to calculate the gene-drug associations. In our revised manuscript, we have included the following paragraph for more detailed explanation of our analyses (Methods section, page 15~16, highlighted in yellow). 

“For gene-drug associations, a list of major cancer-driver alterations, including single nucleotide variations, small insertions and deletions were considered as a predictive genomic biomarker to evaluate drug response interactions. For each drug candidate, drug sensitivity data (AUCs) were analyzed by comparing tumors with the selected genomic alteration to those without using the Wilcoxon rank-sum test. Samples with unknown status of a given alteration were excluded from the analysis. To evaluate lineage-specific drug sensitivities in gynecologic tumors, drug sensitivity data were analyzed by comparing tumors from each pathologic entity to the rest using Wilcoxon rank-sum test. For transcriptome analysis, tumors were classified as “sensitive” (Z-score < -0.5) or “resistant” (Z-score > 0.5) based on drug sensitivity data and subjected to Gene Set Enrichment Analysis (GSEA).”* 

Major Point 4: In the initial study that incorporated 139 gynecological tumor specimens, it seems that the number of each tumor type is quite unbalanced, with 18 cervical cancer samples, 29 uterine/endometrial cancer samples, and 92 epithelial ovarian cancer samples included. How could you manage to guarantee an unbiased analysis among the three types of gynecological cancers with an unbalanced number of tumor samples included? How can you ensure the representativeness of these limited number of samples for each cancer type, especially for cervical cancer and uterine/endometrial cancer? 

*Response 4: We appreciate the reviewer’s insightful comment and fully understand the concern. To evaluate whether our cohort, which mainly consists of ovarian carcinomas could represent the diverse subgroups of gynecologic tumors, we compared the frequency of major cancer-driver alterations in our cohort with TCGA, including ovarian carcinoma, cervical cancer and uterine/endometrial carcinomas. Notably, we found that our cohort constituted comparable levels of major cancer-driver genes in respective cancer types, including TP53 in ovarian carcinoma[2], PIK3CA and PTEN in cervical cancer[3] and PTEN, PIK3CA, ARID1A and CTNNB1 in uterine/endometrial cancer[4]. Furthermore, consistent with previous observation, our lineage-specific drug sensitivity analysis demonstrated significant anti-tumor activity of everolimus in endometrial cancer[5], which further advocates the representativeness of our samples for each cancer type (page 5, highlighted in yellow and additional file 1: Fig. S2).* 

Major Point 5: For figure 5f, the number of ID2 positive patients is too small (n=3). Please enlarge the sample size for analysis. 

*Response 5: We greatly appreciate the reviewer’s important suggestion. In the revised manuscript, we have significantly expanded our retrospective studies on olaparib response with ID2 expression from 13 to 41 cases (ID2-positive, n=17; ID-2 negative, n=24), which further augmented our previous observation. Consistently, we discovered that patients with low ID2 expression showed significantly prolonged survival to olaparib treatment (page 10, highlighted in yellow and Fig. 5F in revised main figures).* 

Major Point 6: Please invite a native speaker to polish the language of the entire manuscript. 

*Response 6: We thank the reviewer’s suggestion and requested a native speaker to polish the language of the entire manuscript.* 

Response to Reviewer #2 

General Remark: In this manuscript, the authors established a library of 139 gynecologic tumors, as well as 66 PDCs. The experimental protocol is largely followed their previous publications, but with different emphasize of cancer types for gynecologic cancers. The pharmacogenomics data exhibits as a good resource, but the analysis and experiments need further improvement. 

*Response: We thank the Reviewer for summarizing our work and providing constructive suggestions to improve our previous manuscript.* 

Major Point 1: It is critical to establish the link between tumor biospecimen and PDCs, as the authors did in their Nature Genetics paper. However, in this manuscript, the evidence is not strong. For example, authors showed the mutational landscape in PDCs, but several PDCs do not have any key mutations at all (Figure 1b). For each key mutation gene, do they have similar mutation frequency in PDC compared to tumor samples? It is unclear how the authors make the comparison in figure 1 c (upper panel). In figure 1c (bottom panel), transcriptomic expression, it is unclear how many samples are involved in this spearman correlation? If only 6 samples are included, why only selected 6 samples? How many genes do they select for this spearman correlation? Furthermore, for ovarian cancer, CNV may be the dominant feature, and the authors may also show the similarity of CNV between PDC and tumor samples. 

*Response 1: We greatly appreciate the reviewer’s insightful comments and suggestions. For the mutational landscape of gynecologic tumors, we have further expanded the list of major cancer-driver genes, including MET, MYC, MYCN, ERBB2, MTOR and NOTCH4. As a result, we found that all tumors harbored at least one key mutation (Fig 1B in revised main figures). 

We apologize for not being explicit in providing detailed explanation behind the mutational comparison between primary tumors and matched patient-derived tumor cells (PDCs) in figure 1c. Figure 1c depicts the frequency of non-synonymous cancer-driver mutations that were observed in primary tissue (black, left axis), PDC (blue, right axis), or both (grey, upper axis). As seen in figure R5, majority of the mutations, including TP53, BRCA2, ERBB3, EGFR, etc. are primarily located within the upper axis, indicating that these genomic alterations are well retained from primary tissues to matched PDCs. 

Furthermore, transcriptomic analysis between primary tissues and matched PDCs were conducted in 6 cases because we only had those samples available at the moment. As a result, we extracted 7947 bona fide tumor-intrinsic genes that showed high correlations (r > 0.6). Lastly, as the reviewer has suggested, we compared the genetic similarity of copy number variations (CNVs) between primary tissue specimens and matched PDCs. Consistently, we found that major cancer-driver CNVs, including FGFR1, CDK4, MDM2 and NF1 were well propagated (Additional file 1: Fig. S4).* 

Major Point 2: TCGA consortium has performed a comprehensive analysis across gynecologic tumors, which covered >2500 samples (Berger et al., Cancer Cell, 2018). The authors should also compare the genomic features of their tissues samples and PDCs with TCGA data. 

*Response 2: We greatly appreciate the reviewer’s suggestion. In the revised manuscript, we compared the frequency of major cancer-driver alterations in our cohort with TCGA, including ovarian carcinoma, cervical cancer and uterine/endometrial carcinomas. Notably, we found that our cohort constituted comparable levels of major cancer-driver genes in respective cancer types, including TP53 in ovarian carcinoma, PIK3CA and PTEN in cervical cancer and PTEN, PIK3CA, ARID1A and CTNNB1 in uterine/endometrial cancer (page 5, highlighted in yellow and additional file 1: Fig. S2).* 

Major Point 3: The authors collected 139 tumor, but only select 66 PDCs for the follow up study? Any reason for this? Most of the PDCs are EOCs, Any reason for this? Are EOCs easier to set up PDCs? E.g., EOCs are easier to culture in EGF medium than any other gynecological tumors? 

*Response 3: Our cohort mainly comprises of EOCs as the patients who visit our hospital with gynecologic tumors are primarily diagnosed with EOCs. Hence, the number of tumor specimens that were included in our study was biased towards EOCs. Furthermore, EOCs had a considerably higher in vitro success rate compared to both cervical and endometrial cancers. Although, uterine sarcomas exhibited a similar success rate to that of EOCs, the total number of patient samples that we received was quite small compared to EOCs.* 

Major Point 4: In figure 2c, the authors should make a pair-wise comparisons among all cancer types. 

*Response 4: We thank the reviewer for the suggestion. We have conducted a pair-wise comparisons among all cancer types in figure 2c and confirmed that our previous observations were consistently identified where ovarian and endometrial cancers demonstrated the highest EGFR and PI3K pathway activities, respectively.* 

Major Point 5: It is unclear for the PDX model validation. For example, the authors compared serous and clear cell in PDC model (figure 3d), while they compared serous/clear cell and control in PDX model (figure 3e/f). My understanding is that the authors want to compare the serous and clear cell, so it is necessary to add clear cell PDX in figure 3e, and add serous carcinoma in figure 3f. 

*Response 5: We appreciate the reviewer’s critical insight and fully understand the reviewer’s concern. However, our results were mainly derived from statistical analysis between serous and clear cell PDCs, suggesting that not every serous tumor would respond to cediranib and vice versa. We were able to demonstrate a representative case of cediranib sensitivity in serous tumor-derived PDX model. While, we could perform additional in vivo experiments using clear cell type PDX models, it may provide artificial results. In such context, the same concern also applies to the in vivo validation of PI3K inhibitor in serous type PDX models. Furthermore, generation of clear cell type PDX models demands extensive time, approximately 6 to 9 months, due to its slow tumor growth rate. Therefore, we expect that full pharmacological validation in vivo will require at least 11 to 12 months, which significantly surpasses the given time of revision. Lastly, the major focus of our manuscript was based on pharmacogenomic interactions of PDCs to facilitate rapid identification of potential therapeutic targets or treatment approaches and PDX experiments were validations of such findings. Therefore, we believe that additional in vivo validations may lie beyond our original scope. However, our preliminary results warrant further investigation, which we plan to conduct in our next research in near future.* 

Major Point 6: In figure 4, the author provide evidence to prove that tumors have TP53 mutation would be more sensitive to olaparib, but the authors may need more evidence. For example, the authors showed only 20-30% reduction on cell viability introduced by BRCA1 and/or TP53 mutants, compared to at least 60% reduction in their NG paper. Furthermore, the authors may need additional cell line to confirm this result. In the discussion, the authors mentioned: "Notably, we demonstrated that addition of TP53 mutation could be a significant molecular determinant for PARP inhibition therapy". Again, the evidence is not strong enough. Importantly, the dosage is quite high, but still not strong effect. Any reason for this? The authors may consider to try different dosages. 

*Response 6: We appreciate the reviewer’s important observation. To address such concern, we have generated various dominant-negative forms of TP53 mutants, including R249S, R273H and R175H. Consistent with our previous observation, cytotoxic activities of olaparib were significantly enhanced on an OVISE ovarian cancer cell line that were transduced with TP53 mutants (page 9, highlighted in yellow and Fig. 4D in revised main figure).* 

Major Point 7: For figure 5d, it is unclear the correlation is Spearman correlation or Pearson correlation. 

*Response 7: We apologize for the oversight. The figure 5d shows Pearson correlation. This has been amended and annotated in the figure legends.* 

Major Point 8: Figure 5f, the sample size is too small for this kind of analysis, especially for ID2 positive samples (n=3). 

*Response 8: We greatly appreciate the reviewer’s important suggestion. In the revised manuscript, we have significantly expanded our retrospective studies on olaparib response with ID2 expression from 13 to 41 cases (ID2-positive, n=17; ID-2 negative, n=24), which further augmented our previous observation. Consistently, we discovered that patients with low ID2 expressions showed significantly prolonged survival to olaparib treatment (page 10, highlighted in yellow and Fig. 5F in revised main figure).* 

Minor Point 1: Multiple references are not correctly cited. For example, the authors sated "We have established a library of .." and cite [26]. However, the reference author list is not the current authors, and the paper is not the paper the authors want to mention. The authors need to correct their reference list to provide clear background. 

*Response 1: We apologize for these mistakes. In the revised manuscript, we have corrected all the citations.* 

Minor Point 2: The p-value format is not correct throughout the manuscript. 

*Response 2: In the revised manuscript, we ensured that all p-value formats are correct.* 

Minor Point 3: There are multiple grammar errors and typos need to be addressed. 

*Response 3: We apologize for multiple grammar errors and typos. We have ensured that all grammar errors and typos are corrected in the revised manuscript.* 

Minor Point 4: Figure 1c lacks the x and y axis label. 

*Response 4: We have revised our figure to show that all x, y and z axes are correctly labeled.* 

Minor Point 5: Figure 5a lacks y axis label for enrichment score. 

*Response 5: We have revised our figure to show that y axis is correctly labeled for enrichment score.* 

Minor Point 6: The y axis of fig 4b is missing. 

*Response 6: We have revised our figure to show that y axis is correctly labeled. 


References 

1. Heo EJ, Cho YJ, Cho WC, Hong JE, Jeon HK, Oh DY, Choi YL, Song SY, Choi JJ, Bae DS, et al: Patient-Derived Xenograft Models of Epithelial Ovarian Cancer for Preclinical Studies. Cancer Res Treat 2017, 49:915-926. 
2. Cancer Genome Atlas Research N: Integrated genomic analyses of ovarian carcinoma. Nature 2011, 474:609-615. 
3. Cancer Genome Atlas Research N, Albert Einstein College of M, Analytical Biological S, Barretos Cancer H, Baylor College of M, Beckman Research Institute of City of H, Buck Institute for Research on A, Canada's Michael Smith Genome Sciences C, Harvard Medical S, Helen FGCC, et al: Integrated genomic and molecular characterization of cervical cancer. Nature 2017, 543:378-384. 
4. Cancer Genome Atlas Research N, Kandoth C, Schultz N, Cherniack AD, Akbani R, Liu Y, Shen H, Robertson AG, Pashtan I, Shen R, et al: Integrated genomic characterization of endometrial carcinoma. Nature 2013, 497:67-73. 
5. Slomovitz BM, Jiang Y, Yates MS, Soliman PT, Johnston T, Nowakowski M, Levenback C, Zhang Q, Ring K, Munsell MF, et al: Phase II study of everolimus and letrozole in patients with recurrent endometrial carcinoma. J Clin Oncol 2015, 33:930-936.*

**Second round of review**

**Reviewer 1**

They have adequately addressed my concerns.

**Reviewer 2**

The reviewer appreciated the significance of the work, and the author addressed most of the comments. However, the authors should carefully address the concerns raised for the original manuscript, including clarifications, labeling, and figure legend. 

1, to author’s response to Major Point 1:
The question: “It is unclear how the authors make the comparison in figure 1 c (upper panel)” is not clearly explained. 
As the author described below, this comparison was based on mutation frequencies of driver mutations in primary tissues and PDCs. How did the authors obtain the distances of each dot to the 0 point of Z axis? In addition, author should explain why some dots are in dark blue while some others are in light blue.

2, to author’s response to Major Point 2:
The comments: “The authors should also compare the genomic features of their tissues samples and PDCs with TCGA data” was addressed partially. 
The author performed analysis between their tissues and TCGA data, but they should also perform the comparison between TCGA gynecologic tumors and PDCs (The same analysis in Fig 1C).

3, to author’s response to Major Point 5:
The comments: “My understanding is that the authors want to compare the serous and clear cell, so it is necessary to add clear cell PDX in figure 3e, and add serous carcinoma in figure 3f” is not clearly explained by the author. 
Considering the authors’ statement that the PDX experiments were validations of such findings of PDCs and the results of Fig. 3E&F are only representative ones, I suggest the authors to revise their statement accordingly. In particularly, discuss the limitation of the results without further PDX model.

4, to author’s response to Major Point 6:
The authors should label the IC50 values in Fig. 4D.

5, to author’s response to Minor Point 2:
The point: “The p-value format is not correct throughout the manuscript” is not addressed by the author. The author did not correct the p-value format in the manuscript. For example, page 12 line 35, P=1.518-06 is incorrect. 

6, to author’s response to Minor Point 4:
The point: “Figure 1c lacks the x and y axis label” is not fully addressed.
These are still some problems of figure labelling. For example, the color key of Figure 1C is incorrect, and the legend of Fig. 3A is incorrect.

7, to author’s response to Minor Point 5:
The point: “Figure 5a lacks y axis label for enrichment score” The authors should also label the name of y axis.

8, to author’s response to Minor Point 6:
The point: “The y axis of fig 4b is missing” – there is still some issues with the y-axis of fig 4b. Not sure if this due to the incompatible system or not, please double check.

**Authors Response**

**Point-by-point responses to the reviewers’ comments:**

Response to Reviewer #1 

General Remark: They have adequately addressed my concerns. 
 *Response: We greatly appreciate the reviewer for acknowledging our contribution to address all the major and minor concerns that were previously raised.* 

Response to Reviewer #2 

General Remark: The reviewer appreciated the significance of the work, and the author addressed most of the comments. However, the authors should carefully address the concerns raised for the original manuscript, including clarifications, labeling, and figure legend. 

*Response: We greatly thank the reviewer for appreciating the significance of our work and our efforts to address most of the comments.* 

Major Point 1: The question: “It is unclear how the authors make the comparison in figure 1 c (upper panel)” is not clearly explained. 
As the author described below, this comparison was based on mutation frequencies of driver mutations in primary tissues and PDCs. How did the authors obtain the distances of each dot to the 0 point of Z axis? In addition, author should explain why some dots are in dark blue while some others are in light blue. 

*Response: We deeply apologize for not clearly explaining figure 1c and causing additional confusions. Figure 1c depicts the frequency of non-synonymous mutations that were either shared between primary tumor tissues and matched PDCs or private to either one. The position of each dot or respective mutation is located on the quadrant based on its shared/private rate between primary tumor tissues and matched PDCs (page 25, Figure legend for Fig. 1C in revised manuscript, highlighted in yellow). For example, we found that BRCA2 mutation was shared between all primary tumor tissues and matched PDCs, thus it has been located directly on the z-axis. On the other hand, TP53 mutation dot is located slightly towards the left quadrant because one patient tumor harbored TP53 mutation but the matched PDC didn’t. The distance of each dot to the 0 point of Z-axis, X-axis or Y-axis is based on the number of patient cases that harbored each respective mutation. As you can see in the figure R1, genomic mutation of TP53 was the most prevalent event. 

Furthermore, some dots are in dark blue because multiple dots have been overlaid on top of one another. Since mutations in BRAF, ARID2, CDKN2A and KDR have the same shared mutation rate between primary tumor tissues and matched PDCs, they have been overlaid on each other. To avoid any further confusions, we have removed any overlaid dots and annotated each mutation next to the respective node (Fig. 1C in revised figures). We hope that our explanation sufficiently clarified any confusion.* 

Major Point 2: The comments: “The authors should also compare the genomic features of their tissues samples and PDCs with TCGA data” was addressed partially. 
The author performed analysis between their tissues and TCGA data, but they should also perform the comparison between TCGA gynecologic tumors and PDCs (The same analysis in Fig 1C). 

*Response: As figure 1C is based on the shared mutation rate between primary tumor tissues and matched PDCs, comparison between TCGA gynecologic tumors and our PDCs will be difficult as they have been derived from different patients. Rather, we performed additional computation analysis to compare the genomic frequency of major cancer driver genes between TCGA ovarian cancers and our ovarian cancer-derived PDCs. Due to the low number of PDCs that were sequenced, we didn’t observe comparable levels of major cancer-driver genes. However, we found that TP53 mutation, the most prevalent somatic mutation in ovarian cancer, has been detected at a similar rate between TCGA and our PDCs.* 

Major Point 3: The comments: “My understanding is that the authors want to compare the serous and clear cell, so it is necessary to add clear cell PDX in figure 3e, and add serous carcinoma in figure 3f” is not clearly explained by the author. 
Considering the authors’ statement that the PDX experiments were validations of such findings of PDCs and the results of Fig. 3E&F are only representative ones, I suggest the authors to revise their statement accordingly. In particularly, discuss the limitation of the results without further PDX model. 

*Response: We thank the reviewer for the suggestion. In the revised manuscript, we noted that PDX experiments were validations of our findings in PDCs and Fig. 3E and F are only representative cases. Furthermore, we discussed the limitation of our results without further PDX models and functional validation are warranted (page 11, discussion section in revised manuscript, highlighted in yellow)* 

Major Point 4: The authors should label the IC50 values in Fig. 4D. 

*Response: We thank the reviewer for the suggestion. In the revised manuscript, we have included the Log (IC50) values in figure 4D (page 9 in revised manuscript, highlighted in yellow).* 

Major Point 5: The point: “The p-value format is not correct throughout the manuscript” is not addressed by the author. The author did not correct the p-value format in the manuscript. For example, page 12 line 35, P=1.518-06 is incorrect. 

*Response: We apologize for this omission in our previous manuscript. In the revised manuscript, we have ensured that all p-values are formatted correctly (Highlighted in yellow in revised manuscript).* 

Major Point 6: The point: “Figure 1c lacks the x and y axis label” is not fully addressed. 
These are still some problems of figure labelling. For example, the color key of Figure 1C is incorrect, and the legend of Fig. 3A is incorrect. 

*Response: We believe there could be some incompatibility issues in terms of images. We have exported the original figures as images and replaced the previous version to ensure that all labels and legends are correctly annotated.* 

Major Point 7: The point: “Figure 5a lacks y axis label for enrichment score” The authors should also label the name of y axis. 

*Response: We thank the reviewer for the suggestion. In the revised manuscript, we have included the labels for y-axis in figure 5a.* 

Major Point 8: The point: “The y axis of fig 4b is missing” – there is still some issues with the y-axis of fig 4b. Not sure if this due to the incompatible system or not, please double check. 

*Response: We believe this could be another incompatibility issue. We have exported the original figure as an image and replaced it to ensure that y-axis is present.*
